# Could Local Application of Hypoxia Inducible Factor 1-α Enhancer Deferoxamine Be Promising for Preventing of Medication-Related Osteonecrosis of the Jaw?

**DOI:** 10.3390/biomedicines11030758

**Published:** 2023-03-02

**Authors:** Gül Merve Yalcin-Ülker, Murat Günbatan, Gonca Duygu, Merva Soluk-Tekkesin, Ceyda Özcakir-Tomruk

**Affiliations:** 1 Oral and Maxillofacial Surgery Department, Faculty of Dentistry, Istanbul Okan University, Istanbul 34947, Türkiye; 2 Oral and Maxillofacial Surgery Department, Faculty of Dentistry, Tekirdag Namık Kemal University, Tekirdag 59030, Türkiye; 3 Department of Tumour Pathology, Institute of Oncology, Istanbul University, Istanbul 34093, Türkiye; 4 Oral and Maxillofacial Surgery Department, Faculty of Dentistry, Yeditepe University, Istanbul 34728, Türkiye

**Keywords:** bisphosphonate, deferoxamine, hypoxia-inducible factor 1-alpha, medication-related osteonecrosis of the jaw, tooth extraction

## Abstract

This experimental study investigates the prophylactic effect of deferoxamine (DFO) on medication-related osteonecrosis of the jaw (MRONJ). Thirty-six female Sprague Dawley rats received zoledronic acid (ZA) for eight weeks to create an osteonecrosis model. DFO was locally applied into the extraction sockets with gelatin sponge (GS) carriers to prevent MRONJ. The specimens were histopathologically and histomorphometrically evaluated. Hypoxia-inducible factor 1-alpha (HIF-1α) protein levels in the extraction sockets were quantified. New bone formation rate differed significantly between groups (*p* = 0.005). Newly formed bone ratios in the extraction sockets did not differ significantly between the control group and the GS (*p* = 1), GS/DFO (*p* = 0.749), ZA (*p* = 0.105), ZA-GS (*p* = 0.474), and ZA-GS/DFO (*p* = 1) groups. While newly formed bone rates were higher in the ZA-GS and ZA-GS/DFO groups than in the ZA group, the differences were not significant. HIF-1α levels differed significantly between groups (*p* < 0.001) and were significantly higher in the DFO and ZA-GS/DFO groups than in the control group (*p* = 0.001 and *p* = 0.004, respectively). While HIF-1α levels were higher in the ZA-GS/DFO group than in the ZA group, the difference was not significant. While HIF-1α protein levels and new bone formation rate were elevated in the DFO-treated group, the effect was not significant. Further large-scale studies are needed to understand DFO’s preventative effects on MRONJ and the role of HIF-1α in MRONJ pathogenesis.

## 1. Introduction

Bisphosphonate (BP) compounds are commonly used to reduce complications such as hypercalcemia and pathological fractures due to various malignant tumors by inhibiting bone osteoclastic activity, improving quality of life, and reducing pain in cancer patients with bone metastases. Intravenous BP therapy is effective in correcting hypercalcemia due to malignancy, and in treating tumors associated with breast, prostate, and lung cancers, and metastatic osteolytic lesions due to multiple myeloma [1,2]. The American Association of Oral and Maxillofacial Surgeons (AAOMS) describes BP-related osteonecrosis of the jaw (BRONJ) as a clinical condition characterized by the formation of exposed bone areas in the maxillofacial region that persist for ≥8 weeks in patients who have used or are still using BP-based drugs, and have not received radiotherapy to the head and neck region [3].

In recent years, BRONJ has been identified as a potential complication of nitrogen-containing BP (NBP)-based drugs, especially when administered intravenously. Since these clinical lesions can also be caused by antiresorptive drugs, such as denosumab and sunitinib, and antiangiogenetic drugs, such as bevacizumab, AAOMS has named this condition medication-related osteonecrosis of the jaw (MRONJ). AAOMS recently recognized other medications causing MRONJ, including fusion proteins (aflibercept), mammalian target of rapamycin (mTOR) inhibitors (everolimus), selective estrogen receptor modulators (raloxifene), immunosuppressants (methotrexate and corticosteroids), and antiresorptives prescribed for osteoporosis (romosozumab) [3].

While it remains unknown why BPs cause osteonecrosis, especially in the jaw, theories for MRONJ pathophysiology include changes in bone remodeling, excessive suppression of bone resorption, inhibition of angiogenesis, synergy between bone micro-fractures due to continuous microtrauma and periodontal bacterial invasion, suppression of innate or acquired immunity, vitamin D deficiency, toxicity, and inflammation or infection in soft tissues [3]. Studies have also shown that BPs inhibit vascular endothelial growth factor (VEGF) [4,5,6].

Previous experimental and clinical studies have shown that promoting angiogenesis is an important treatment strategy in the healing and regeneration of all tissues, including bone. Deferoxamine (DFO), which is indicated in the treatment of iron poisoning by the US Food and Drug Administration (FDA), has been shown in many studies to support bone healing, especially of irradiated bones [7,8,9]. DFO contributes to angiogenesis by inhibiting the prolyl-hydroxylase enzyme. It was observed that DFO causes iron chelation in the callus region in fracture healing models. In addition, it was observed that iron accumulation inhibits the prolyl-hydroxylation of hypoxia-inducable factor 1-alpha (HIF-1α), causing it to accumulate.

Elevated HIF-1α protein levels cause its nuclear translocation and dimerization with hypoxia-inducible factor 1-beta (HIF-1β), inducing the expression of VEGF and other mediators involved in neovascularization [10,11]. DFO has been shown to improve neoangiogenesis and neoangiogenesis via this mechanism when administered by local injection between bone fragments in fracture or distraction osteogenesis in experimental animal studies, facilitating osteogenesis [8,9,11].

In the current literature, there are experimental studies aiming at preventing MRONJ by inducing VEGF. In a study conducted by Tamari et al. (2019), they hypothesized that injection of endothelial progenitor cells to the surrounding soft tissue might stimulate MRONJ-like lesions [12]. Another study conducted by Sharma et al. (2021) on the effect of local delivery of hydrogel encapsulated VEGF for the prevention of medication-related osteonecrosis of the jaw has been investigated. They concluded that the application of locally delivered VEGF into the extraction sockets might induce bone healing and prevent MRONJ via a pro-angiogenic and immunomodulatory mechanism [13]. In general, the main aim of these studies was preventing MRONJ via a neoangiogenesis mechanism. Considering that the primary triggering factor of MRONJ is tooth extraction and the drugs that cause MRONJ formation are prescribed more and more each day, materials that are simpler to prepare for use in routine oral surgery practice and cheaper in terms of accessibility are needed.

Given the importance of vascularization inhibition via VEGF in MRONJ pathophysiology, these results suggest that the local application of an agent such as DFO to promote neoangiogenesis and healing by inducing VEGF could be protective against MRONJ after tooth extraction. This study investigates the effects of local application of DFO with a gelatin sponge (GS) carrier, which is used in daily oral surgery practice, on bone healing and neovascularization, and its protective effects against MRONJ in healthy and zoledronic acid (ZA)-treated rats.

## 2. Materials and Methods

### 2.1. Animal Care and Procedures

This study used 36 12-week-old female Sprague Dawley rats with an average weight of 200 ± 25 g from Yeditepe University Experimental Animals Research Center (YÜDETAM; Istanbul, Türkiye) and the study was approved by the Yeditepe University Experimental Animals Local Ethics Committee (protocol number 2020-845). Rats were randomly divided into six groups. The dosage and treatment duration of ZA injection was as described by Dayisoylu et al. [14]. Rats in groups I (control), II (GS), and III (GS/DFO) were given intraperitoneal (IP) sterile saline (SS) at 0.1 mg/mL three times a week for eight weeks, starting on the first day of the study. Rats in groups IV (ZA), V (ZA-GS), and VI (ZA-GS/DFO) were given IP ZA at 0.1 mg/kg (Zometa [4 mg/5 mL]; Novartis, Istanbul, Türkiye) three times a week for eight weeks, starting on the first day of the study. The procedures applied to rats in each group are shown in Table 1.

At the end of the eighth week, general anesthesia was induced by intramuscular injection of 80–100 mg/kg ketamine hydrochloride (Ketasol; Richterpharma, Wels, Austria) and 10 mg/kg 2% xylazine hydrochloride (Rompun; Bayer, Türkiye) as both analgesic and anesthetic before tooth extraction under the supervision of a veterinarian. Bacitracin and neomycin sulfate ointment (Thiocillin [5 g]; Abdi İbrahim, Istanbul, Türkiye) was applied to the rat’s eyes to prevent ophthalmic complications during general anesthesia. Rats were prepared for surgery under aseptic and antiseptic conditions while under general anesthesia, and right upper first, second, and third molars were extracted.

The extraction sockets of rats in groups I (control) and IV (ZA) were left empty, and extraction socket healing was observed under normal and ZA-treated conditions. GSs (Surgifoam Gelatin Sponge; Ethicon, Johnson & Johnson, Raritan, NJ, USA) were cut to socket size and inserted into the extraction sockets of rats in groups II (GS) and V (ZA-GS). GSs cut to socket size and saturated with DFO (Desferal [0.5 g/7.5 mL]; Novartis) were inserted into the extraction sockets of rats in groups III (GS/DFO) and VI (ZA-GS/DFO). GS placement was fixed with 4.0 biodegradable sutures (Vicryl Rapide [polyglactin 910]; Ethicon, Johnson & Johnson, Raritan, NJ, USA).

The amount of DFO used to saturate GSs was calculated according to the volume of the rat extraction socket. In our previous study using the same physical conditions, it was observed that the mean socket bone volume following extraction of the lower left second molars of rats was 2.5834 ± 0.46697 mm^3^ under normal conditions (control group, equivalent to group I) and 2.6347 ± 0.2583 mm^3^ in ZA-treated rats (ZA group, equivalent to group IV) eight weeks after tooth extraction. A mean mandibular molar extraction socket volume was calculated based on these findings and was 2.60905 mm^3^ (~2.6 mm^3^) [15]. Given the extraction of 3 molars, the average volume was estimated to be 7.8 mm^3^, and a solution with a volume of 7.8 mm3 (7.8 µL) was prepared. The desired DFO concentration in the solution was calculated based on the dose used by Donneys et al. for pathological mandibular fractures (200 μg/300 μL) [9]. In this study, the DFO dose applied to the extraction socket was 5.2 μg/7.8 μL. Commercially available DFO (Desferal; Novartis, Istanbul, Türkiye) is sold as 0.5 g of sterile lyophilized powder in a 7.5 mL vial. When the ratios were compared, it was found that Donneys et al. used a solution with the same DFO concentration as the commercially available volume. Therefore, a 0.5 mg/7.5 mL solution containing lyophilized DFO powder and sterile distilled water was prepared and dropped on the sterile GS at a dose of 5.2 μg/7.8 μL with a sterile pipette tip.

All experimental rats were euthanized by decapitation eight weeks after tooth extraction and sixteen weeks after the start of the study (Table 1). Then, the maxilla of each rat was dissected and removed and placed in 10% phosphate-buffered neutral formaldehyde for histopathological, histomorphometric, and immunohistochemical evaluation. Tissues were kept in this solution for two weeks for fixation.

### 2.2. Histological Evaluation

#### 2.2.1. Histopathological and Histomorphometric Examination

Dissected rat maxillae were kept in a prepared decalcification solution containing 50% formic acid and 20% sodium citrate. The solutions were changed once a week, and the decalcification process was completed after 30 days. After decalcification, sagittal sections covering the entire defect area were dissected. Paraffin blocks prepared from routinely processed decalcified specimens were cut into 4 μm slices and stained with hematoxylin and eosin (H&E). The stained sections were examined by a researcher blinded to the groups with an Olympus BX60 microscope connected to a computer with a color video camera (Tokyo, Japan). All measurements for histomorphometric analysis were made with the Olympus Image Analysis System 5. Images were taken at different magnifications via the camera, transferred to the computer screen, and calibrated. The histopathological presence of inflammation, foreign body reactions, and necrotic tissues was assessed. Inflammation was scored as 0 (absent), 1 (mild), 2 (moderate), and 3 (severe) according to its intensity. In the histomorphometric examination, all socket (total bone [TB]) and vital bone (VB) areas were measured. The rate of new bone formation was calculated using these data:

New bone formation rate = (VB/TB) × 100

#### 2.2.2. Immunohistochemical Staining and Evaluation

The paraffin blocks were cut serially into ~5 μm thick sections on charged slides for immunohistochemistry. Firstly, the sections were penetrated and dried overnight in an autoclave, then deparaffinized with xylene for 30 min, washed with 99% alcohol for 15 min, followed by 96% alcohol and distilled water. The Histostain-Plus Bulk Kit (Zymed Laboratories, South San Francisco, CA, USA) was used for analysis. The sections were microwaved four times for 5 min in a citrate buffer for antigen retrieval. Endogenous peroxidase activity was blocked by incubating the sections with 3% hydrogen peroxide before washing with distilled water and phosphate-buffered saline for 5 min. Non-specific reactions were prevented by incubating sections with a blocking solution. Sections were incubated with a 1:50 dilution of the anti-HIF-1α primary antibody (GeneTEX, Irvine, CA, USA) for 120 min, followed by the secondary antibody for 25 min. The reaction was visualized using the chromogen 3-amino-9-ethylcarbazole (Zymed Laboratories, South San Francisco, CA, USA). Finally, the sections were counterstained with Mayer’s hematoxylin, coverslipped, and evaluated under a light microscope. A semiquantitative score system was used to evaluate immunostaining data:

0 (−): 0–10% staining immunopositivity
1 (+): 10–25% staining immunopositivity
2 (++): 25–50% staining immunopositivity
3 (+++): 50–70% staining immunopositivity
4 (++++): >75% staining immunopositivity

### 2.3. Statistical Evaluation

The data were analyzed using the Statistical Package for Social Sciences (SPSS) for Windows v.23 (IBM, Armonk, NY, USA). The normality of the data was assessed using the Shapiro–Wilk test. The chi-squared test was used to compare the inflammation and necrosis scores by group, and multiple comparisons were performed with a Bonferroni-correction Z-test. The histomorphometric analysis was statistically evaluated using a one-way analysis of variance to compare normally distributed data by group, and multiple comparisons were performed with the Tukey’s Honest Significant Difference test. The Kruskal–Wallis test was used to compare non-normally distributed data by group. Non-normally distributed immunohistochemical staining intensity scores were compared between groups using the Kruskal–Wallis test, and multiple comparisons were performed with Dunn’s test. All results are presented as mean ± standard deviation and median (minimum–maximum). All results with *p* < 0.05 were considered statistically significant.

## 3. Results

### 3.1. Histopathological Analysis

**Figure 1:**
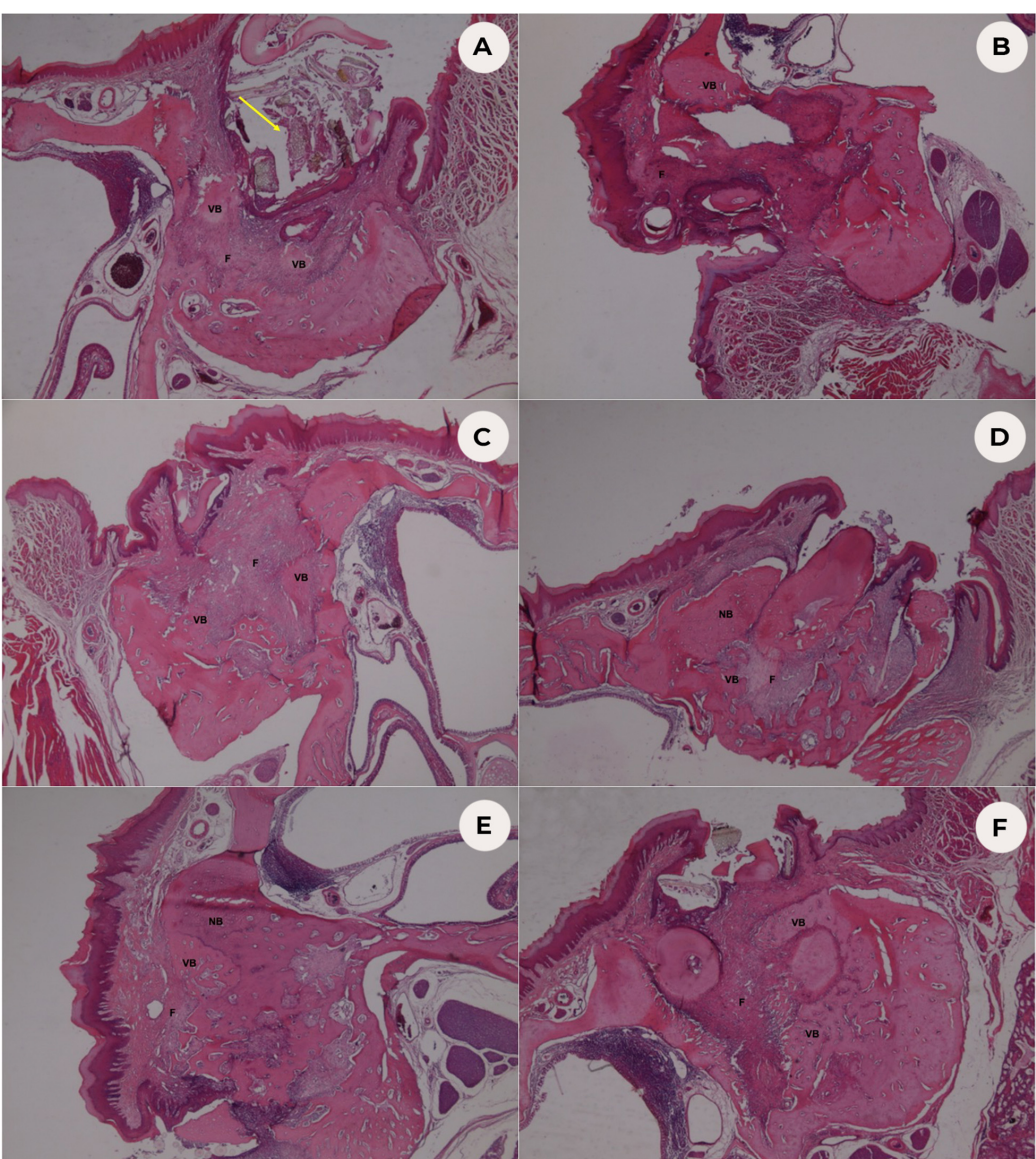
(**A**) Histological image of Group I (Control): new bone formation and fibrosis were observed in the extraction socket under the epithelium on which food residues accumulated (arrow). VB: Vital Bone; F: Fibrosis. (**B**) Histological image of Group II (GS): new bone formation, fibrosis, and mild inflammation were seen in the extraction socket. VB: Vital Bone; F: Fibrosis. (**C**) Histological image of Group III (GS/DFO): new bone formation and fibrosis filled the extraction socket. VB: Vital Bone; F, Fibrosis. (**D**) Histological image of Group IV (ZA): failure in healing of the bone and necrotic bone in the extraction socket. NB: Necrotic Bone; VB: Vital Bone; F: Fibrosis. (**E**) Histological image of Group V (ZA-GS): a small necrotic portion of the alveolar bone surrounding the extraction socket. NB: Necrotic Bone; VB: Vital Bone; F: Fibrosis. (**F**) Histological image of Group VI (ZA-GS/DFO): new bone formation and fibrosis were detected in the extraction socket. VB: Vital Bone; F: Fibrosis. (Hematoxylin & Eosin, Original magnification ×40).

Histopathological evaluations of extraction sockets are shown in Table 2, and histological images of H&E stained experimental groups are shown in Figure 1. The distributions of inflammatory responses did not differ significantly between groups (*p* = 0.108), and foreign body reactions were not observed in any group. However, necrotic area distributions did differ significantly between groups (*p* < 0.001). In groups I (control), II (GS), and III (GS/DFO), necrosis was not observed. There were necrotic areas in all specimens in group IV (ZA). Necrosis was present in 66.7% of specimens in group V (ZA-GS) and 33.3% in group VI (ZA-GS/DFO).

### 3.2. Histomorphometric Analysis

Newly formed bone rates in the extraction sockets were determined (Table 3) and differed significantly between groups (*p* = 0.005). They did not differ significantly between group I (Control) and groups II (GS; *p* = 1), III (GS/DFO; *p* = 0.749), IV (ZA; *p* = 0.105), V (ZA-GS; *p* = 0.474), and VI (ZA-GS/DFO; *p* = 1). However, they did differ significantly between group III (GS/DFO) and groups IV (ZA; *p* = 0.004) and V (ZA-GS; *p* = 0.037). While newly formed bone rates in groups V (ZA-GS) and VI (ZA-GS/DFO) were higher than in group IV (ZA), the difference was not significant (*p* = 0.946 and *p* = 0.193, respectively).

### 3.3. Immunohistochemical Analysis

**Figure 2:**
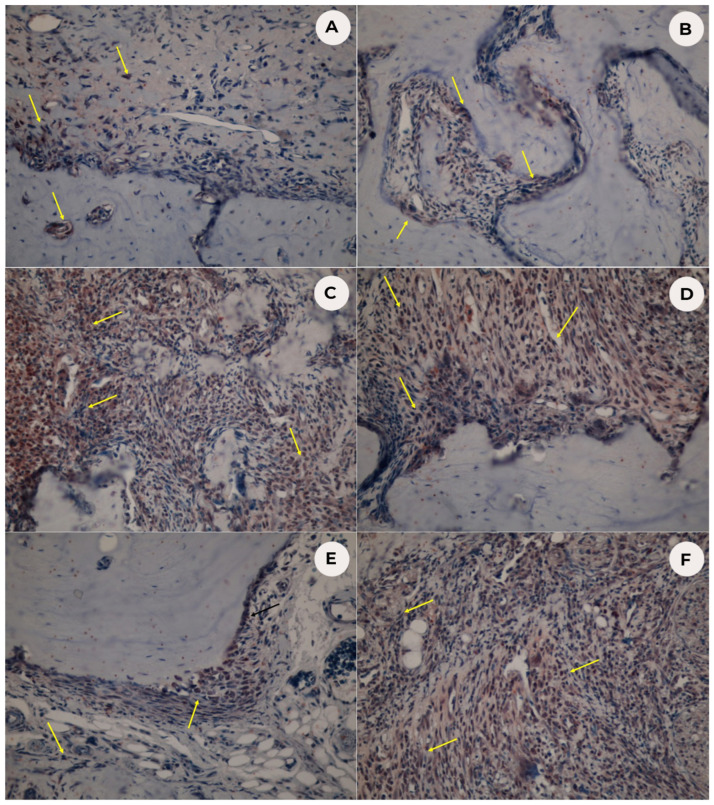
Representative histological images of HIF-1α immunohistochemical staining of control and experimental groups (**A**) Group I (Control): a few positive cells were shown (arrows). (**B**) Group II (GS): positive cells were shown around the bone trabecula (arrows). (**C**) Group III (GS/DFO): diffuse positivity was observed (arrows). (**D**) Group IV (ZA): positive cells were seen in both bone periphery and connective tissue (arrows). (**E**) Group V (ZA-GS): focal positive cells were determined (arrows). (**F**) Group VI (ZA-GS/DFO): positive cells were observed in large areas (arrows). (HIF-1α, Original magnification ×400).

Microscopic images of HIF-1α immunohistochemical staining are shown in Figure 2, and the median intensity scores for HIF-1α protein in the extraction sockets are shown in Table 4. HIF-1α protein levels differed significantly between groups (*p* < 0.001). HIF-1α intensity scores in group I (Control) did not differ significantly from those in groups II (GS; *p* = 1), IV (ZA; *p* = 0.442), and V (ZA-GS; *p* = 1). However, those of DFO groups III (DFO) and VI (ZA-GS/DFO) were significantly higher than those of group I (control; *p* = 0.001 and *p* = 0.004, respectively). In addition, HIF-1α intensity scores differed significantly between group II (GS) and DFO groups III (DFO; *p* = 0.005) and VI (ZA-GS/DFO; *p* = 0.011). However, the scores did not differ significantly between groups IV (ZA) and V (ZA-GS; *p* = 1). Moreover, while the scores were higher in group VI (ZA-GS/DFO) than in group IV (ZA), the difference was not significant (*p* = 1). Finally, HIF-1α intensity scores did not differ significantly between groups V (ZA-GS) and VI (ZA-GS/DFO; *p* = 0.442).

## 4. Discussion

With increasing life expectancy and various direct and indirect treatment modalities targeting bone and surrounding structures, modern clinicians have to cope with the side effects and complications of these drugs, including MRONJ. While some accepted MRONJ treatment strategies exist in the literature, none are entirely evidence-based. Furthermore, the systemic condition and host responses of patients taking these drugs vary. Therefore, preventing such complications is more logical than coping with them.

The most important factor in physiological or pathological wound healing processes is ensuring the adequate transportation of required defense cells, growth factors, cytokines, and progenitor cells to the affected area. The success of this process depends on the area’s adequate vascularization or sufficient neoangiogenesis. Angiogenesis is the formation of new blood vessels during endothelial cell growth, differentiation, and migration. During this mechanism, signaling molecules such as VEGF, the protein primarily inducing and regulating vascular growth, must bind to receptors on endothelial cells [16,17]. It has been shown that inhibiting angiogenesis is effective in causing MRONJ [3].

It is widely believed that BPs have antiangiogenic properties and suppress VEGF production via apoptosis [18,19]. NBPs such as ZA directly inhibit angiogenesis in vitro and in vivo, reducing vascularity in MRONJ lesions and quantitatively decreasing microvessels during early bone healing stages [5,6,13,20,21]. Furthermore, angiogenesis in post-extraction socket healing is inhibited by BPs, and both BPs and denosumab led to decreased arterial area, venous area, and overall vascularity in periodontal tissues during early and late MRONJ development [22]. These data suggest that microcirculation disorder in the lesion area may be an important contributor to MRONJ formation.

VEGF and transforming growth factor-β1 (TGF-β1) are synergistically effective in the MRONJ mechanism. While low-dose BPs increase osteoblast proliferation in the early stage, they reduce the cells’ differentiation capacity, resulting in damage to bone quality [23]. Manzano-Monero et al. (2018) reported that low-dose BP is effective by increasing levels of molecules such as TGF-β1 and VEGF, which affect cell growth, and decreasing levels of molecules such as bone morphogenetic protein 2 (BMP2) and receptor activator of nuclear factor kappa-Β ligand (RANKL), which are necessary for cell maturation [24,25,26]. Therefore, it can be hypothesized that VEGF inhibition is very important for MRONJ pathogenesis due to its direct and indirect effects.

Some studies have explored the importance of neovascularization of the region in treating and preventing MRONJ, both for inflammatory response regulation and growth factor migration to the region. One of the most important of these are the autologous platelet concentrates (APCs), frequently used for regenerative purposes in oral and maxillofacial surgery. APCs include growth factors such as platelet-derived growth factor (PDGF), TGF-β1, VEGF, and epidermal growth factor EGF [27,28,29]. APCs have local effects by being applied in combination with surgical treatments. These platelet-rich preparations accelerate tissue healing and bone regeneration [29]. The main APC role in tissue healing, which involves growth factor release in the necrotic bone area, is the stimulation of tissue healing through cell chemotaxis, proliferation, and differentiation, angiogenesis, and new bone matrix deposition [30].

APCs are classified based on leukocyte and fibrin content. Of these, platelet-rich plasma (PRP) and platelet-rich fibrin (PRF) are frequently used to treat MRONJ [27,28,29]. In addition, studies are reporting that PRF, which is placed and fixed in the socket following the tooth extraction, significantly reduces early complications when tooth extraction is planned in patients using antiresorptive drugs such as BP or denosumab [31]. Additionally, there are clinical reports supporting the curative effect of PRF in combination with photobiomodulation for MRONJ [32]. It has been shown that photobiomodulation has a contributing effect on new bone formation, and organization of deposition of collagen [33].

The disadvantage of using these autologous products is that they cannot maintain long-term stability. It has been reported that PRFs maintain stability for 3 to 7 days, depending on the method [34]. VEGF must be at a certain level for four weeks to stabilize endothelial cells in newly formed vessels. However, VEGF’s very short half-life precludes the effective use of its recombinant protein, either experimentally or clinically [35,36,37]. Therefore, the indirect induction of neoangiogenesis appears to be a more effective approach for maintaining recovery.

Physiologically, VEGF production is induced by HIF-1α. HIF-1α is one of four subunits of an αβ heterodimeric transcription factor called HIF (HIF-1α, HIF-2α, HIF-3α, and HIF-1β) that is active in hypoxic environments [38,39]. Wang and Semenza (1993) first suggested that DFO, an iron chelator agent, could induce HIF-1α activity [38]. DFO is a chelating agent used to treat iron poisoning and hemochromatosis. It causes the induction of HIF-1α expression, inducing the production of VEGF and other angiogenic factors [40]. It has been reported that DFO contributes to osteogenic and angiogenic responses in bone in surgical procedures targeting new bone formation, such as distraction osteogenesis applied to long bones [41].

Farberg et al. (2012) investigated the effect of DFO on radiation-induced hypovascularity and impaired bone healing in rats through distraction osteogenesis in irradiated jaws [8]. Examination with a microcomputed tomography angiography method found high neovascularization in the distraction osteogenesis spaces of the rat jaws treated with DFO. Furthermore, they observed new bone formation between the irradiated bone fragments of all DFO-treated rats. In addition, Donneys et al. (2013) investigated the healing effect of DFO injections into the space between fracture fragments in jaws reverted to a pathological healing pattern by radiation [9]. They observed that when DFO was applied in samples where pathological healing was expected, the healing was supported, and neoangiogenesis, a prerequisite for a healthy recovery, was realized in 42%.

Chung et al. (2013) found that DFO applied to human periodontal ligament cells induced osteoblastic activity and mineralization via the mitogen-activated protein kinase (MAPK), nuclear factor κB (NF-κB), and nuclear erythroid 2-related factor-2/antioxidant response element pathways [42]. Furthermore, Jia et al. (2016) reported that DFO does not affect mesenchymal stem cell proliferation in osteoporotic rats. However, it induced the expression of angiogenetic factors by inducing osteogenic differentiation and upregulating mRNA in mesenchymal stem cells [43].

Furthermore, some studies have reported that DFO promotes healing by inducing neoangiogenesis in tissues other than bone. Bonham et al. (2018) investigated pressure sores in diabetic rats and found that local DFO injections into the area may have a healing effect [44]. Another study on mature diabetic rats showed that DFO could regulate recovery by contributing to neovascularization in diabetic elderly rats [45]. A study by Sinder et al. (2018) investigated post-surgical radiotherapy treatment of breast cancer patients who had resective surgery by atomic force microscopy, showing that topical DFO affected collagen fibril organization and wound healing. It was concluded that DFO could eliminate the effects of radiation both macroscopically and microscopically in the areas where it was applied [46].

In this study, while GS increased new bone formation in the extraction sockets of non-ZA-treated rats, the difference was not significant. Similarly, DFO-saturated GS increased new bone formation in the healthy extraction sockets, but the difference was not significant. Nevertheless, immunohistochemical analysis of these groups showed significantly elevated HIF-1α protein levels in extraction sockets in group III (GS/DFO) compared with the control group (*p* = 0.004). We aimed to investigate the effect of DFO on MRONJ rat models, and performed histological evaluations eight weeks after tooth extraction in rats to evaluate the late phase of healing in extraction sockets. Consequently, the elevated HIF-1α protein levels might indicate the accelerating DFO effect on the early phase of extraction socket healing. However, this study’s findings are insufficient to discuss this effect, and further studies are needed to explore this phenomenon.

When the new bone formation rate was evaluated in ZA-treated groups, the effect was similar to the control groups. While GS increased new bone formation in the extraction sockets, the difference was not significant. Similarly, DFO-saturated GS increased new bone formation in the extraction socket of ZA-treated rats, but the difference was not significant. However, there was also no significant difference between the control group and the DFO-applied experimental group (VI). Furthermore, immunohistochemical analyses showed that ZA treatment increased HIF-1α protein levels compared with the control group. However, this increase was only significant between the control and DFO-treated experimental group (VI). The findings of the studies investigating the effect of ZA on HIF-1α protein levels are controversial. Minegaki et al. (2018) reported that hypoxic HIF-1a protein levels were unaffected by ZA-treatment [47].

Other studies have explored the possible connection between MRONJ pathophysiology and the HIF-1α/VEGF pathway. Ge et al. (2016) showed that ZA dose-dependently inhibited cell viability, migration, adhesion, and tube formation by decreasing VEGF expression and secretion. Here, ZA decreased HIF-1α protein levels but did not affect HIF-1α mRNA levels and promoter activity. In addition, they found that ZA decreased HIF-1α protein stability by reducing the activation of the phosphatidylinositol-3-kinase (PI3K)/protein kinase B (AKT)/mTOR and MAPK pathways [48]. Controversially, Trebec-Reynolds et al. (2010) investigated differences in signaling pathways between large and small osteoclasts. They found that VEGF-A mRNA and protein levels were elevated in large osteoclasts, found mostly in MRONJ and periodontitis specimens, compared to small osteoclasts, and that this increase was regulated by HIF-1α, whose mRNA levels were induced by RANKL-mediated activation of NF-κB [49].

We suggest that our findings could reflect our chosen experimental model in which teeth were extracted after eight weeks of ZA treatment. Some studies have explored the effect of DFO on the inflammatory process. Oses et al. (2017) used adipose tissue-derived mesenchymal stem cells (AdMSC) pre-conditioned with DFO under in vitro conditions to investigate the expression of specific factors and cytokines [50]. They reported that DFO increases the expression of proinflammatory cytokines such as interleukin (IL)-4 and IL-5 to indirectly increase the expression of proangiogenic factors such as VEGF and angiopoietin 1 by inducing HIF-1α. Another study used AdMSCs pre-conditioned with DFO and applied to the RAW 264.7 cells to examine DFO’s effect on macrophage polarization [51]. They concluded that DFO might have an immunomodulatory role by inducing macrophage polarization at the M2 phase. In addition, Hellwig-Bürgel et al. (2005) reported that HIF-1α is closely related to immune reactions due to its key mediatory role [52]. Studies on inflammation in MRONJ pathogenesis have recently started to be performed. Numerous studies have also reported that disrupted healing is caused by an insufficient or excessive inflammatory response [21]. Paschalidi et al. (2021) studied osteonecrotic tissue debrided from 30 post-operative patients with MRONJ, classifying M1 and M2 macrophages according to MRONJ stage using the immunofluorescence method [53]. They found that patients with early-stage MRONJ shifted toward M2 macrophages, while patients with advanced-stage MRONJ shifted toward M1 macrophages. They concluded that regulating macrophage function could be important in MRONJ treatment strategies.

In this study, we histopathologically examined inflammation with a semiquantitative method. We found that DFO did not contribute significantly to inflammation in the groups in which it was applied (*p* = 0.108). We believe this might be due to the suture material applied to fix the GS in the socket in the GS-applied groups affecting the inflammatory response. We applied DFO locally into the extraction sockets. Previous studies on DFO are either in vitro or animal-based. Several studies used distraction osteogenesis in irradiated jaws to examine the healing of the pathological fracture line, locally injecting DFO into the affected area [8,9]. They investigated a carrier molecule since the extraction sockets of rats and humans are not enclosed spaces, and it is not clinically feasible to inject DFO into the extraction socket. Another study by the same group examined pathological fracture healing in irradiated jaws using an implantable hyaluronic acid (HA–DFO) conjugate, finding that the healing properties of the HA–DFO conjugate were observed in 91% of experimental pathological fractures [11].

We believe that conjugating DFO with an agent known to contribute to regeneration, such as HA, might be a very useful approach for increasing regeneration capacity and facilitating the local application of agents that promote healing, such as DFO, especially when a carrier is required [54]. However, using such conjugated agents requires significant investigation, and the difficulties associated with their production and storage in routine oral surgical applications such as tooth extraction are a significant disadvantage. Therefore, we opted to use GS, an inexpensive and accessible agent used in oral surgery practice.

While gelatin is the product of partial hydrolysis of natural collagen, it is used as a dressing material in clinical applications of tissue engineering applications and sponge form in drug delivery systems due to its non-toxic and non-carcinogenic properties, biocompatibility, and biodegradability [55]. Gelatin can be prepared in a spongy form suitable for tissue engineering applications. The porous 3D structure of GS scaffolds can provide multiple spaces for cell adhesion [55]. The mechanical properties of GS are improved using elements such as colloidal silver and gold nanoparticles, and chemicals such as antibiotics, collagen, transglutaminase, glutaraldehyde, and chitosan to enhance its anti-inflammatory and antibacterial properties [55,56,57]. In addition, it has been observed that GS can be used as a dressing material in conjugate form, or by absorbing the agent to facilitate healing.

In this study, a commercially available GS for routine oral surgery use known to contain only gelatin was chosen because it is believed that any additional material would make it difficult to understand the effect of DFO alone. While calculating the amount of DFO to be used, the concentration used by Donneys et al. (2013) in pathological mandibular fractures was used as its basis with the average extraction socket volume. The major limitation of this study was that it was unclear how many days the DFO-saturated GSs biodegraded, and how many days these materials continued to release DFO in the extraction sockets. Further studies are needed to understand this in more detail and provide scientific, evidence-based support for using GS, a cheap and practical material used in oral surgery practice, combined with drugs that enhance wound and bone healing, such as DFO.

## 5. Conclusions

This is the first experimental study investigating the prophylactic effect of local DFO application on MRONJ. GSs saturated with DFO were applied to the extraction sockets of rats following eight weeks of ZA treatment. While elevated HIF-1α protein levels and new bone formation were observed in the DFO-treated group, the effect was not significant. However, the absence of a significant effect on new bone formation rate but a significant effect on HIF-1α protein levels between the DFO-saturated GS and control groups suggests that local DFO application might be prophylactic for MRONJ after tooth extraction. Further studies are needed with more specimens to understand this effect. In addition, molecular studies are required to understand the importance of the RANKL/NF-κB/HIF-1α/VEGF pathway on pathological and inflammatory bone loss and MRONJ in the context of the DFO HIF-1α inducer. Such studies will be useful both for understanding the possible prophylactic and therapeutic effects of DFO, and for a deeper understanding of MRONJ pathophysiology.

## Figures and Tables

**Table 1:** Procedures applied to rats in the control and experimental groups weekly. Abbreviations: SS: Sterile Saline; ZA: Zoledronic Acid; GS: Gelatin Sponge; DFO: Deferoxamine; IP: Intraperitoneal.

	SS	ZA
	(0.1 mg/kg 3 Times per Week IP)	(0.1 mg/kg 3 Times per Week IP)
Weeks		
1	Groups I, II, and III	Groups IV, V, and VI
2	Groups I, II, and III	Groups IV, V, and VI
3	Groups I, II, and III	Groups IV, V, and VI
4	Groups I, II, and III	Groups IV, V, and VI
5	Groups I, II, and III	Groups IV, V, and VI
6	Groups I, II, and III	Groups IV, V, and VI
7	Groups I, II, and III	Groups IV, V, and VI
8	Groups I, II, and III	Groups IV, V, and VI
MAXILLARY MOLAR EXTRACTION		
Procedure		
Empty Socket Group I (Control) Group IV (ZA)	GS Group II (GS) Group V (ZA-GS)	GS/DFO Group III (GS/DFO) Group VI (ZA-GS/DFO)
16	EUTHANASIA	

**Table 2:** Comparison of inflammation and necrosis by groups.

	Group I	Group II	Group III	Group IV	Group V	Group VI	Test Statistics	*p* *
	Control	GS	GS/DFO	ZA	ZA-GS	ZA-GS/DFO		
Inflammation								
Absent	2 (33.3)	5 (83.3)	3 (50)	1 (16.7)	1 (16.7)	4 (66.7)	22	0.108
Mild	3 (50)	1 (16.7)	2 (33.3)	2 (33.3)	2 (33.3)	2 (33.3)		
Moderate	1 (16.7)	0 (0)	1 (16.7)	1 (16.7)	3 (50)	0 (0)		
Severe	0 (0)	0 (0)	0 (0)	2 (33.3)	0 (0)	0 (0)		
Necrosis								
No	6 (100) ^a^	6 (100) ^a^	6 (100) ^a^	0 (0) ^b^	2 (33.3) ^ab^	4 (66.7) ^ab^	24	<0.001
Yes	0 (0) ^a^	0 (0) ^a^	0 (0) ^a^	6 (100) ^b^	4 (66.7) ^ab^	2 (33.3) ^ab^		

* Pearson’s Chi-Squared Test; ^a,b^: There is no difference between groups with the same letter.

**Table 3:** Comparison of the new bone formation ratio values of the extraction sockets.

	New Bone Formation Rate (%)	
	Mean ± SD	Median (Minimum–Maximum)
Group I Control	43.57 ± 18.66 ^ab^	46.2 (16.3–62.9)
Group II GS	44.49 ± 12.05 ^ab^	41 (31.3–60.3)
Group III GS/DFO	54.15 ± 16.95 ^b^	50.4 (37.3–84)
Group IV ZA	22.57 ± 6.22 ^a^	24.2 (12.5–28.6)
Group V ZA-GS	29.48 ± 7.22 ^a^	29.6 (20.3–36.6)
Group VI ZA-GS/DFO	41.12 ± 14.74 ^ab^	38.4 (25–68)
	F = 4.269	
*p*	0.005	

F: One way ANOVA; Kruskal–Wallis Test; ^a,b^: There is no difference between groups with the same letter.

**Table 4:** Comparison of the intensity levels of HIF-1α expression in the extraction sockets.

	Mean ± SD	Median (Minimum–Maximum)	Test Statistics	*p* *
Group I (Control)	1.33 ± 0.52	1.00 (1.00–2.00) ^a^	27.117	<0.001
Group II (GS)	1.50 ± 0.55	1.50 (1.00–2.00) ^a^		
Group III (GS/DFO)	3.50 ± 0.55	3.50 (3.00–4.00) ^b^		
Group IV (ZA)	2.50 ± 0.55	2.50 (2.00–3.00) ^ab^		
Group V (ZA-GS)	2.17 ± 0.41	2.00 (2.00–3.00) ^ab^		
Group VI (ZA-GS/DFO)	3.33 ± 0.52	3.00 (3.00–4.00) ^b^		

* Kruskal–Wallis Test; ^a,b^: There is no difference between groups with the same letter.

## Data Availability

The datasets generated during and/or analyzed during the current study are available from the corresponding author on reasonable request.
